# Hemodynamic Assessment and Transcatheter Intervention Treating Pulmonary Vein Stenosis

**DOI:** 10.1016/j.jscai.2024.102250

**Published:** 2024-08-10

**Authors:** Ashish H. Shah, Triston Eastman, Petra Jenkins

**Affiliations:** a Section of Cardiology, Department of Internal Medicine, Max Rady College of Medicine, University of Manitoba, Winnipeg, Manitoba, Canada; b Department of Internal Medicine, University of Manitoba, Winnipeg, Manitoba, Canada; c Adult Congenital Heart Disease Services, Department of Cardiology, Liverpool Heart and Chest Hospital NHS Trust, Liverpool, United Kingdom

**Keywords:** atrial fibrillation ablation, hemodynamics, pulmonary vein stenosis, transcatheter interventions

A 66-year-old woman started experiencing progressive dyspnea on physical activity 18 months after undergoing radiofrequency pulmonary vein (PV) isolation for paroxysmal atrial fibrillation. CT pulmonary angiography during the levo phase demonstrated significant stenosis of the left upper lobe vein, whereas the left lower lobe vein could not be visualized. Cardiac catheterization demonstrated a mean right atrial pressure of 12 mm Hg, pulmonary artery pressure of 33/18 with a mean of 24 mm Hg, mean pulmonary capillary wedge pressure in the right lung of 16 mm Hg, and 25 mm Hg in the left lung. The left pulmonary angiogram demonstrated severe stenosis in the left upper lobe pulmonary vein (LUPV) during the levo phase.

After a transseptal puncture, we could cannulate the LUPV using a balanced middleweight coronary angioplasty wire. A 4F glide catheter was placed beyond the ostium and its position into a true lumen was confirmed. Simultaneous pressure in the PV and the left atrium (LA) demonstrated ∼6 mm Hg gradient (Figure 1A). Doppler signal during transesophageal echocardiography demonstrated 1.8 m/s velocity and persistent forward flow through the LUPV (Figure 1B), whereas the left lower lobe pulmonary vein could not be visualized. Despite multiple attempts, we could not cannulate the left lower lobe pulmonary vein. After initial dilatation using 2.5-mm and 3.0-mm compliant balloons, we exchanged a Balance Middle Weight wire (Abbott) for an Amplatz extra stiff wire. The LUPV was further dilated using 5.0 × 20 and 8.0 × 20 mm peripheral angioplasty balloons up to 8 atm, lasting for ∼40 to 60 seconds (Figure 1C, D). With sequential balloon dilation, the PV grew; however, within 10 minutes after ballooning the PV ostium appeared to be getting stenosed again with an increasing gradient between the LUPV and the LA (Figure 1E). Hence, we stented this LUPV using a Genesis 8 mm × 18 mm stent (Figure 1F). The stent was further postdilated using an 8 mm diameter noncompliant balloon up to 8 atm that resolved any gradient between the LUPV and the LA (Figure 1G). Moreover, poststenting, the Doppler velocity across the LUPV decreased to 0.8 m/s (Figure 1H). The patient was advised to continue with rivaroxaban long-term. Repeat CT pulmonary angiography 7 years postprocedure demonstrated patent stent.

**Figure 1:**
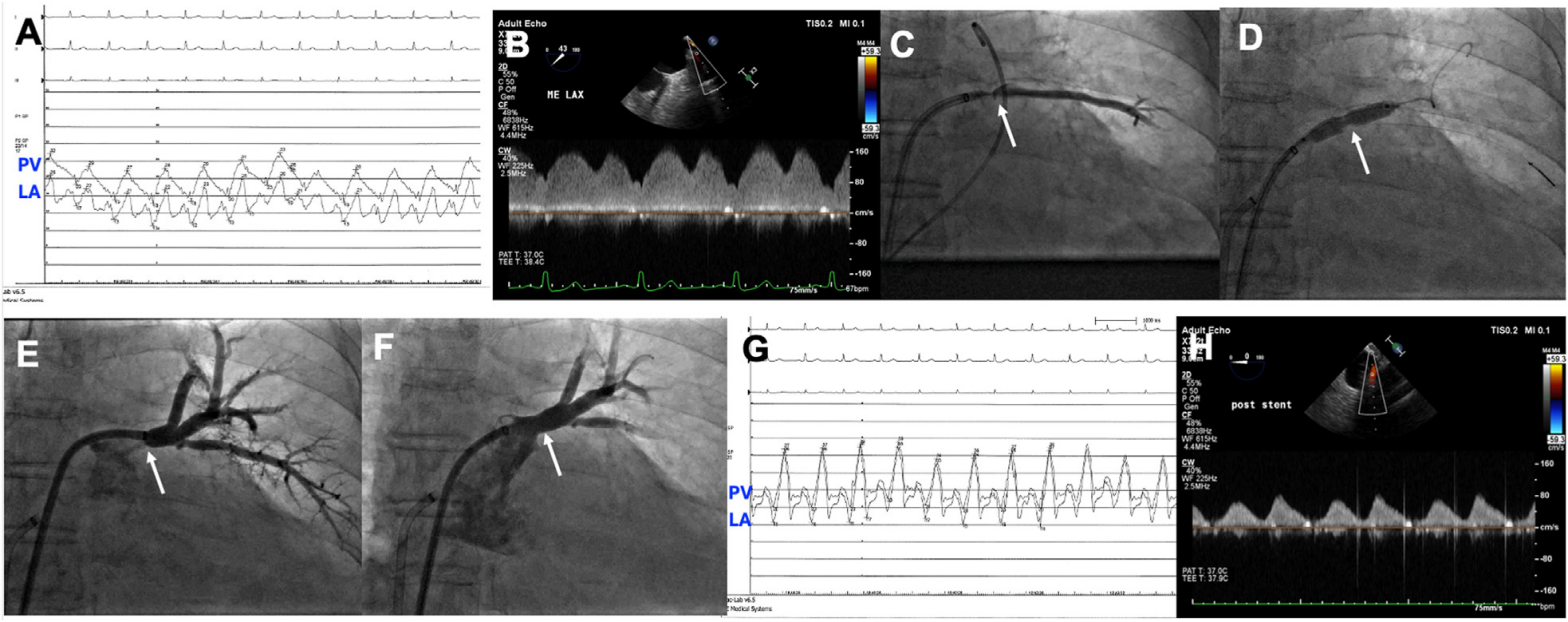
**Pulmonary vein (PV) stenosis evaluation and intervention.** (**A**) Simultaneous pressure in the PV (loss of the typical phasic flow) and the left atrium (LA), (**B**) left upper lobe pulmonary vein (LUPV) Doppler flow characteristic preintervention, (**C**, **D**) ballooning of the LUPV, (**E**) restenosis of the PV ostium after ballooning, (**F**) poststenting of LUPV ostium, (**G**) simultaneous pressure in PV and LA, poststenting, and (**H**) LUPV Doppler flow characteristic poststenting.

PV stenosis (PVS) post–atrial fibrillation ablation is not uncommon, and the incidence increases with repeated procedures.^1^ Only the patients with severe stenosis involving more than 1 PV are likely to be symptomatic.^2^ Significant PVS is defined as the mean gradient between the PV and LA of >3 mm Hg, loss of phasic flow, and decreased pulsatility on Doppler examination.^3^ CT pulmonary venogram and ventilation perfusion scan can help define the PVS severity and their hemodynamic impact.^3^ Transcatheter intervention treating a stenosed PV is the treatment of choice, whereas cannulating an occluded PV as in this patient remains challenging, and symptomatic patients may warrant surgical lobectomy. Stenting offers a better result than ballooning only; however, at a 3-year follow-up post-PV intervention, nearly one-third of the patients were noted to have >75% stenosis. Hemoptysis, pericardial tamponade, and PV perforation are the most encountered procedural complications.^3^

## Pearls in Hemodynamics

• Differential pulmonary capillary wedge (PCW) pressures across lung fields or a significant pressure gradient between PCW pressures and LV end-diastolic pressures should raise suspicion for pulmonary vein stenosis.
• The classic finding in severe pulmonary vein stenosis is an elevated mean pulmonary vein pressure (or PCW pressure), with a pulmonary vein to a left atrial gradient of >3 mm Hg and loss of phasic flow.
• Interpreting the severity of pulmonary vein stenosis should consider the degree of angiographic narrowing, measured gradients across the stenoses, and differential flow to each lobe, as the flow may affect gradients.
